# Early Intervention in Orbital Floor Fractures: Postoperative Ocular Motility and Diplopia Outcomes

**DOI:** 10.3390/jpm12050671

**Published:** 2022-04-22

**Authors:** Cherng-Ru Hsu, Lung-Chi Lee, Yi-Hao Chen, Ke-Hung Chien

**Affiliations:** 1 Department of Ophthalmology, Tri-Service General Hospital, National Defense Medical Center, Taipei 11490, Taiwan; josephinesheu@gmail.com (C.-R.H.); kidday0205@gmail.com (L.-C.L.); doc30879@ndmctsgh.edu.tw (Y.-H.C.); 2 Department of Medical Science, Graduate School, National Defense Medical Center, Taipei 11490, Taiwan

**Keywords:** orbital floor fracture, early intervention, ocular motility, diplopia

## Abstract

Purpose: Orbital floor fractures commonly occur during orbital trauma. Currently, the indications for orbital fracture repair and the appropriate duration between trauma and surgical intervention remain controversial. Methods: Eyes diagnosed with orbital floor fractures that underwent reconstruction surgery were retrospectively reviewed. Demographic data were analyzed. Patients were classified based on the timing of the surgical intervention after injury. Ocular limitation and diplopia were evaluated preoperatively and postoperatively at one week, one month, and three months. Results: Two hundred seventy eyes of 270 patients (174 males and 96 females, mean age: 40.9 ± 16.3 years) were identified. The mean duration from injury to surgical intervention was 18.0 ± 21.2 days (range: 0–117 days). In the subgroup analysis, compared to delayed treatment, the early repair of floor fractures, i.e., within 7 days, was associated with significant motility and diplopia resolution at one week (*p* = 0.001, *p* < 0.001), one month (*p* < 0.001, *p* < 0.001), and three months (*p* < 0.001, *p* < 0.001). Sex and the duration from injury to repair were significantly associated with postoperative ocular motility (*p* = 0.001; *p* = 0.024) and diplopia (*p* < 0.001; *p* = 0.008) at three months. Multivariate analysis revealed that preoperative limitation and diplopia were correlated with postoperative limitation (*p* = 0.007) and diplopia (*p* = 0.001), respectively. Conclusions: The duration between orbital floor fracture and surgical treatment was associated with postoperative limitation and diplopia. Our results suggest that earlier intervention in symptomatic patients with orbital trauma may improve postoperative visual function.

## 1. Introduction

Orbital bone fractures commonly occur during midface trauma, as the medial wall and floor are weak points in the facial skeleton [1]. Blowout fractures caused by blunt trauma lead to a sudden rise in intraocular pressure, wall fractures, and an intact orbital rim [2]. Extraocular muscle entrapment and ischemic change, trapping, or prolapse of the orbital soft tissue may result in ocular motility limitations, diplopia, and visual dysfunctions. In addition, esthetically debilitating conditions, such as enophthalmos or globe displacement, may appear with large bony defects [3].

The surgical interventions commonly performed to repair orbital fractures include the transconjunctival preseptal approach with or without the transcaruncular, transcutaneous, or endoscopic (transmaxillary or transnasal) approaches with the insertion of thin alloplastic implants. Currently, the indications for orbital fracture repair and the appropriate duration between trauma and surgical intervention remain controversial [4]. Immediate surgery is required for trapdoor fractures that cause a severe oculocardiac reflex, which mostly occurs in children, to prevent morbidity [5,6]. Traditionally, delayed surgical repair within two weeks after trauma is proposed, and a thorough ophthalmic examination can be performed and precise globe positioning can be evaluated after the periorbital edema subsides [7]. Persistent diplopia in the primary gaze, enophthalmos > 2 mm, and fractures with over 50% floor involvement are generally considered indications for reconstruction [8]. However, adhesion and fibrotic scarring around the soft tissue may contribute to technical challenges in delayed treatment and may impede the recovery of ocular motility and visual function.

The aim of this study was to investigate visual function outcomes, including diplopia and ocular motility, after surgical repair for orbital floor fractures by comparing different intervention times after fracture.

## 2. Methods

This study was approved by the Institutional Review Board (No.: B202105114) of the Tri-Service General Hospital (TSGH) in Taipei, Taiwan, and conducted in accordance with the tenets of the Declaration of Helsinki.

In this observational case series, we retrospectively reviewed the medical records of patients diagnosed with an orbital floor fracture who received surgical repair in a single tertiary referral hospital over a 10-year period. The surgeries for orbital fracture repair were performed by two oculoplastic surgeons (C.-R.H. and K.-H.C.). The enrollment criteria were as follows: (1) patients who had a traumatic orbital floor fracture and underwent orbital reconstruction at TSGH between January 2011 and September 2020; (2) patients who underwent complete ophthalmic perioperative examinations, including preoperatively and postoperatively at one week, one month, and three months; and (3) patients with no history of ocular diseases that cause strabismus or amblyopia. Patients were excluded from the study if they met the following criteria: (1) failure to receive orbital floor fracture repair due to major trauma, unstable vital signs, or loss of vision during traumatic episodes; (2) incomplete perioperative exam records; (3) extraocular muscle entrapment and a significant oculocardiac reflex that underwent immediate surgery based on the traditional criteria; and (4) pediatric patients (under 18 years of age at presentation) with floor fractures. Demographic data, including age, sex, laterality, integrity of the medial wall, diplopia before and after intervention, ocular motility, timing of the surgical intervention, and surgery time, were collected.

External eye photography of the nine cardinal positions of gaze was performed by clinicians for patients with clear consciousness before and after the operation. Ocular limitation before the operation was defined as having pain in a certain direction of extraocular movement (EOM) or an EOM limitation that was confirmed by a forced duction test without the oculocardiac reflex. The limitations before and after the operation are described as having at least one direction of EOM that shows, on the photos, a limitation in comparison to the same direction in the other eye, and diplopia before and after the operation is defined as subjectively reporting double vision in at least one direction of gaze, irrespective of severity. Improvement was defined as the postoperative resolution of limitations or diplopia compared with the preoperative characteristics.

### 2.1. Surgical Procedures

Either titanium mesh or porous polyethylene implants (Medpor sheet; Porex Surgical Inc., College Park, GA, USA) were used for repairing the orbital wall fractures in all patients. A forced duction test was performed at the beginning of surgery. A transconjunctival preseptal approach was used, and lateral canthotomy and inferior cantholysis were routinely performed during surgery to widen the surgical field. If the orbital floor fracture was combined with a medial wall fracture, a medial extension or a transcaruncular approach was used to expose the fracture site. The repositioning of the prolapsed orbital soft tissue, the release of incarcerated muscle, and the insertion of the reconstruction material were performed with care to ensure no tissue herniation. The forced duction test was repeated to confirm free movement without traction at the end of surgery. Periosteum fixation of the lower eyelid was secured before the completion of the surgery.

### 2.2. Statistical Analysis

SPSS statistical software version 26 (SPSS Inc., IBM Company, Chicago, IL, USA) was used to conduct statistical analyses. One-way ANOVA was used for subgroup analysis, and post hoc analysis with Bonferroni’s test was conducted if *p* < 0.05. The Kruskal–Wallis test was performed to compare ocular limitations and diplopia postoperatively. The Wilcoxon signed rank test was used to detect differences between the variables before and after the operation. The Mann–Whitney U test was used to compare differences in ocular motility and diplopia between early and delayed repair. Variables predicting postoperative limitations and diplopia were identified using logistic regression analyses. A *p* value < 0.05 was considered statistically significant.

## 3. Results

### 3.1. Patient Characteristics and Demographic Data

In this study, 270 eyes of 270 patients (174 men and 96 women) were enrolled. The duration from injury to surgical intervention was determined and divided into seven groups. There were 45 (16.7%) patients who received surgery within 24 h, 37 (13.7%) who received surgery within 3 days, 31 (11.5%) who received surgery within 1 week, 39 (14.4%) who received surgery within 2 weeks, 33 (12.2%) who received surgery within 3 weeks, 38 (14.1%) who received surgery within 30 days, and 47 (17.4%) who received surgery after 1 month. Among the 47 (17.4%) patients who received surgery after 1 month, 14 (29.8%) subjects were delayed due to personal reasons (poor socioeconomic status or low health literacy), 20 (42.5%) patients were delayed due to the transfer time from other hospitals, and 13 (27.7%) patients were delayed due to the need to stabilize medical comorbidities. The mean age of the patients was 40.67 ± 17.15 years. A significantly younger age (33.69 ± 13.87, *p* = 0.034) was observed in patients who received repair within one day. A total of 153 eyes (56.7%) combined with medial wall fractures were confirmed by reviewing the computed tomography (CT) scans. EOM limitation in at least one direction before the operation was found in 140 eyes (51.9%), and there were 123 eyes (45.56%) with diplopia preoperatively. Subgroup analysis showed that, of the 45 patients who received surgery within 24 h, 43 (95.6%) had tissue entrapment confirmed by the forced duction test at the beginning of the operation, and 27 (60.0%) had diplopia, which was significantly more than in other groups (*p* < 0.001, *p* = 0.034, respectively). The mean operation time was 70.06 ± 40.44 min (Table 1).

**Figure 1:**
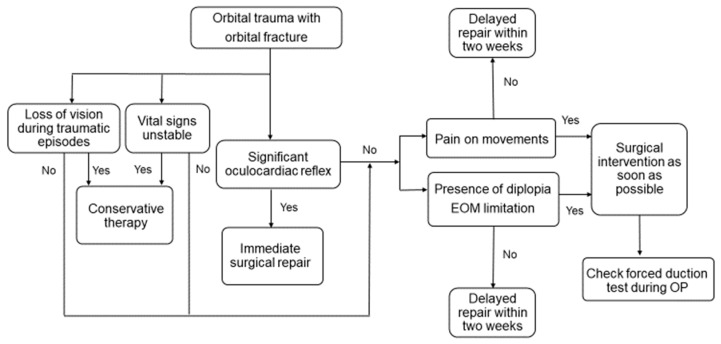
Algorithm for the early management of a blowout fracture. Abbreviations: EOM, extraocular movement; OP, operation.

The flowchart for the early management of an orbital floor fracture is outlined in Figure 1. Initially, trauma with a suspected orbital floor fracture is confirmed by orbital CT. The assessment addresses life-threatening issues by transferring patients to receive conservative therapy and appropriate care if they have unstable vital signs, altered mental status, or loss of vision during traumatic episodes. Next, the presence of a significant nonresolving oculocardiac reflex is determined, and the orbital CT scans are checked again for the possibility of a trapdoor fracture, which is an indication for immediate surgical repair. A complete ophthalmic examination and an evaluation of the nine cardinal positions of gaze are performed. Pain on EOM, the presence of diplopia, and EOM limitation are considered representative of tissue incarceration, and surgical intervention is performed as soon as possible if there are no contraindications. Patients without symptoms receive the traditional repair. The forced duction test is performed at the beginning of the operation, and the outcome is recorded with the degree of limitation.

### 3.2. Surgical Intervention Time and Postoperative Motility and Diplopia

The postoperative EOM limitations at 1 week, 1 month, and 3 months were significantly different among subgroups (*p* = 0.027, *p* < 0.001, and *p* < 0.001, respectively), with 8.89% (*n* = 4), 0%, and 0% of patients who received the earliest surgical intervention observed to have the lowest proportion of postoperative limitations at 1 week, 1 month, and 3 months, respectively. For double vision after the operation, eyes that underwent repair within 24 h had a significantly lower proportion of subjective diplopia than other groups at 1 week (11.11%, *p* < 0.001), 1 month (4.44%, *p* < 0.001), and 3 months (0%, *p* < 0.001) (Table 2).

**Figure 2:**
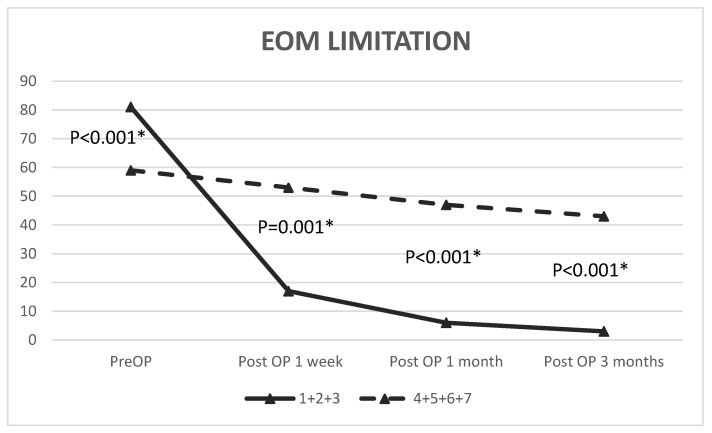
Comparison of EOM limitations between groups that received an operation within one week (1, 2, 3) and after more than one week (4, 5, 6, 7). The Mann–Whitney U test was used to compare between group (1 + 2 + 3) and group (4 + 5 + 6 + 7); EOM, extraocular movement; OP, operation; * *p* < 0.05.

**Figure 3:**
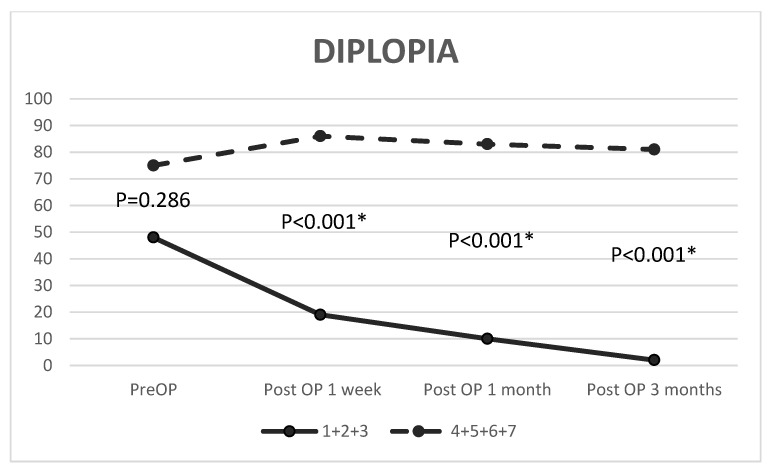
Comparison of diplopia between groups that received surgery within one week (1, 2, 3) and after more than one week (4, 5, 6, 7). The Mann–Whitney U test was used to compare between group (1 + 2 + 3) and group (4 + 5 + 6 + 7); OP, operation; * *p* < 0.05.

Significant improvements in postoperative EOM limitations were found after 1 week (*p* < 0.001), 1 month (*p* < 0.001), and 3 months (*p* < 0.001) in eyes receiving surgery within one week, and the improvement rates were 79.01%, 92.59%, and 96.30%, respectively. In addition, the ocular motility in patients who underwent surgery beyond one week improved significantly by 1 week (*p* = 0.031), 1 month (*p* = 0.001), and 3 months (*p* = 0.001) after the operation, and the rate of motility improvement was 10.17%, 20.34%, and 27.12%, respectively (Figure 2, Table 3). Compared with preoperative measurements, the postoperative measurements indicated improvements of 60.42%, 79.17%, and 95.83% at 1 week (*p* < 0.001), 1 month (*p* < 0.001), and 3 months (*p* < 0.001), respectively. For patients who underwent surgery after more than one week, diplopia did not improve significantly by 1 week (*p* = 0.281), 1 month (*p* = 0.496), or 3 months (*p* = 0.677) postoperatively (Figure 3, Table 4). In addition to nonresolving diplopia, persistent new-onset diplopia was demonstrated in 7% (*n* = 6) of patients at 3 months postoperatively, while two patients had persistent diplopia in vertical gaze at the final visit.

### 3.3. Ocular Motility and Diplopia before and after Repair

The preoperative ocular limitation was significantly greater (*p* < 0.001) in eyes receiving surgery within 7 days. Significant differences existed at 1 week, 1 month, and 3 months postoperatively between patients who underwent surgery after less than one week and those who underwent surgery after more than one week (*p* = 0.001, *p* < 0.001, and *p* < 0.001, respectively). With respect to diplopia, no significant difference (*p* = 0.286) was found before reconstruction between eyes receiving surgery within one week and those receiving surgery beyond one week. However, a significantly higher proportion of diplopia was reached by 1 week, 1 month, and 3 months postoperatively in cases that underwent surgery after more than one week compared to those who underwent surgery within one week (*p* < 0.001, *p* < 0.001, *p* < 0.001, respectively) (Figure 2 and Figure 3).

### 3.4. Variables Associated with Postoperative Limitation and Diplopia

In the univariate analysis, the duration from injury to operation (OR = 1.023, *p* < 0.001), sex (OR = 2.611, *p* = 0.015), and preoperative limitation (OR = 2.412, *p* = 0.011) were significantly associated with postoperative EOM limitation, and the association remained significant in the multivariate regression analysis (OR = 1.030, *p* = 0.001; OR = 2.720, *p* = 0.024; OR = 2.883, *p* = 0.007, respectively). Postoperative diplopia at 3 months was significantly associated with the duration from injury to operation (OR = 1.047, *p* < 0.001), operation time (OR = 1.011, *p* = 0.018), sex (OR = 2.840, *p* = 0.001), and preoperative diplopia (OR = 3.042, *p* = 0.001) in the univariate analysis. However, only the duration from injury to operation (OR = 1.091, *p* < 0.001), sex (OR = 3.246, *p* = 0.008), and preoperative diplopia (OR = 2.774, *p* = 0.001) were significant in the multivariate analysis (Table 5). In our cohort, neither age nor coexistent medial wall fracture was associated with persistent limitation and diplopia at the last follow-up visit.

## 4. Discussion

There are currently controversies regarding the treatment of patients with an orbital floor fracture, mostly with respect to the optimal surgical intervention time and whether to use a conservative or surgical approach [2,4,9,10]. In our relatively large case series study, we demonstrate that the sooner the repair, the better the postoperative visual function improvement with respect to the recovery of ocular motility and diplopia in orbital trauma. Furthermore, we propose an algorithm for the assessment of early intervention for patients with orbital floor fractures that may help to reduce postoperative EOM limitation and diplopia.

Previous research has reported that surgical intervention within two weeks following floor fracture is recommended in symptomatic diplopia with a positive forced duction test or a large floor fracture (>50% floor area) causing latent or significant enophthalmos (>2 mm posterior displacement) [8]. Recently, Yamanaka et al. [11] demonstrated that floor fractures with incarcerated tissue that were repaired within 8 days after injury had better ocular movement than those repaired after 8 days. Kasaee and colleagues found that surgery for blowout fractures should be performed within 4.5 days after trauma to prevent long-term diplopia and EOM limitations [12]. Matteini et al. suggested surgical repair within 3 days of injury in children and within 7 days in adults to avoid permanent postsurgical diplopia [13]. Furthermore, early intervention decreases the risk of developing fibrosis of the impinged orbital tissues that might result in chronic diplopia [14]. In our study, the earliest intervention group was found to have the lowest incidence of limited motility and diplopia in the postoperative period, which may indicate optimal recovery of visual function. However, it has been reported that surgery more than 6 weeks after injury can still achieve improvement of diplopia in the primary gaze [15]. For patients in our cohort who received surgery after more than one week and less than two weeks, EOM limitation achieved significant improvement at 1 month and 3 months postoperatively, while diplopia persisted at each follow-up visit (Supplementary Materials). Of those without diplopia at the initial presentation, eight (2.96%) had induced double vision in a certain direction of gaze. Moreover, the proportion of eyes with postoperative diplopia was even higher than the preoperative condition, especially in eyes that received surgery after more than three weeks, which may correlate with muscle injury or adjoint tissue fibrosis that caused only partial recovery of muscle function despite motility improvements after reconstruction.

In our study, the duration from injury to reconstructive surgery (in days) was significantly associated with EOM limitation and diplopia at the 3-month visit. Meanwhile, our results showed that a repair performed within one day was associated with the lowest incidence of EOM limitation and diplopia after surgery. Together, these findings suggest that the sooner the surgery, the better the recovery of visual function. Older age has been regarded as a risk factor for persistent diplopia postoperatively in previous research [14]. However, age revealed no significant difference in postoperative ocular motility or diplopia in our cohort. The relatively younger age (40.67 ± 17.15) of our patients may account for the better results with respect to regaining muscle function after injury. In contrast to Kasaee et al. [12], who found that combined medial and inferior wall fractures were associated with a higher incidence of EOM limitation than inferior wall fractures alone, our results showed that the coexistence of medial wall fractures was not a significant predictor of postoperative limitations. Although subgroup analysis revealed a trend of a longer operation time in patients with a delayed intervention, the operation time was not significant in the multivariate analysis for limitations or diplopia 3 months after surgery. Tahiri et al. [16] reported that patients with preoperative diplopia had a 9.91times greater risk of persistent diplopia after repair. Similarly, postoperative EOM limitation and diplopia were significantly associated with limitation and diplopia before surgery in our analysis.

There were several limitations in this study. First, due to its retrospective and nonrandomized nature, selection bias may exist due to the inclusion of patients from a tertiary referral center. Second, we did not use the Hess screen test to quantify the degree of diplopia and muscle deviation. Instead, we used subjectively reported symptoms of double vision, which is more easily applied in clinical scenarios during follow-up. Furthermore, the postoperative follow-up was only 3 months in our study. A longer follow-up would be ideal for evaluating the recovery of diplopia and motility, but most patients had generally good functional outcomes, so follow-up beyond clinical necessity was not available. Future investigations with longer patient follow-ups are warranted to determine the final outcomes of postoperative diplopia and EOM limitation.

In conclusion, we propose the early management of orbital floor fractures guided by clinical symptoms and CT images. While the optimum timing of surgery continues to be an area of debate, our results demonstrate that early reconstruction may improve postoperative visual function and promote the recovery of ocular motility and diplopia in orbital trauma.

## Figures and Tables

**Table 1:** Demographic data and characteristics of patients with orbital floor fractures based on different groups of surgical intervention timing.

Group	1	2	3	4	5	6	7	Total	*p* Value
Duration from injury to surgical intervention (days)	0–1	2–3	4–7	8–14	15–21	22–30	31~		
Number of patients (N, %)	45 (16.7%)	37 (13.7%)	31 (11.5%)	39 (14.4%)	33 (12.2%)	38 (14.1%)	47 (17.4%)	270	<0.001 ^#^
Gender (M, %)	30 (66.7%)	30 (81.1%)	15 (48.4%)	26 (66.7%)	24 (72.7%)	23 (60.5%)	26 (55.3%)	174 (64.4%)	0.086 ^$^
Age (mean) (SD)	33.69 (13.87)	40.66 (15.01)	45.24 (19.96)	44.73 (16.79)	41.65 (18.67)	40.79 (19.10)	41.25 (16.05)	40.67 (17.15)	0.034 ^#,‡,†^
Laterality (R, %)	30	22	19	19	13	17	18	138	0.056 ^$^
With medial wall fracture (N, %)	21 (46.7%)	18 (48.6%)	16 (51.6%)	17 (43.6%)	24 (72.7%)	26 (68.4%)	31 (66.0%)	153 (56.7%)	0.043 ^$^
EOM limitation before OP (N, %)	43 (95.6%)	22 (59.5%)	16 (51.6%)	14 (35.9%)	14 (42.4%)	15 (39.5%)	16 (34.0%)	140 (51.9%)	<0.001 ^$^
Diplopia before OP (N, %)	27 (60.0%)	12 (32.43%)	9 (29.03%)	14 (35.90%)	18 (54.55%)	17 (44.74%)	26 (55.32%)	123 (45.56%)	0.034 ^$^
OP time(min) (SD)	65.36 (30.90)	68.89 (54.10)	64.06 (36.19)	66.69 (29.41)	60.27 (40.81)	81.45 (35.30)	82.32 (46.37)	70.06 (40.44)	0.001 ^#,^*^,§^

EOM, extraocular movement; M, male; OP, operation; SD, standard deviation; ^#^ One-way ANOVA, Bonferroni test if *p* < 0.05; ^$^ Chi-square and Fisher’s exact test. ^‡^ Post hoc analysis: *p* = 0.037, between groups 1 and 3; ^†^ Post hoc analysis: *p* = 0.043, between groups 1 and 4; Post hoc analysis: * *p* = 0.027, between groups 5 and 6; ^§^ Post hoc analysis: *p* = 0.009, between groups 5 and 7.

**Table 2:** Comparison of postoperative ocular limitations and diplopia.

Group	Limitation First Week (N, %)	Limitation 1 Month (N, %)	Limitation 3 Months (N, %)	Diplopia First Week (N, %)	Diplopia 1 Month (N, %)	Diplopia 3 Months (N, %)
1	4 (8.89%)	0 (0.00%)	0 (0.00%)	5 (11.11%)	2 (4.44%)	0 (0.00%)
2	7 (18.92%)	2 (5.41%)	1 (2.70%)	8 (21.62%)	4 (10.81%)	1 (2.70%)
3	6 (19.35%)	4 (12.90%)	2 (6.45%)	6 (19.35%)	4 (12.90%)	1 (3.23%)
4	12 (30.77%)	7 (17.95%)	4 (10.26%)	17 (43.59%)	16 (41.03%)	14 (35.90%)
5	13 (39.39%)	12 (36.36%)	11 (33.33%)	18 (54.55%)	16 (48.48%)	16 (48.48%)
6	13 (34.21%)	13 (34.21%)	13 (34.21%)	20 (52.63%)	20 (52.63%)	20 (52.63%)
7	15 (31.91%)	15 (31.91%)	15 (31.91%)	31 (65.96%)	31 (65.96%)	31 (65.96%)
*p* value	0.027 *	<0.001 *	<0.001 *	<0.001 *	<0.001 *	<0.001 *

Kruskal–Wallis test, * *p* < 0.05.

**Table 3:** The relationship of EOM limitation before and after the operation.

Group	Limitation					
	Before OP vs. Post-OP 1 Wk		Before OP vs. Post-OP 1 M		Before OP vs. Post-OP 3 M	
	Improvement Rate (%)	*p* Value	Improvement Rate (%)	*p* Value	Improvement Rate (%)	*p* Value
1 + 2 +3	79.01%	<0.001 *	92.59%	<0.001 *	96.30%	<0.001 *
4 + 5 + 6+ 7	10.17%	0.031 *	20.34%	0.001 *	27.12%	0.001 *

Wilcoxon signed rank test, * *p* < 0.05.

**Table 4:** The relationship of diplopia before and after the operation.

Group	Diplopia					
	Before OP vs. Post-OP 1 wk		Before OP vs. Post-OP 1 m		Before OP vs. Post-OP 3 m	
	Improvement Rate (%)	*p* Value	Improvement Rate (%)	*p* Value	Improvement Rate (%)	*p* Value
1 + 2 + 3	60.42%	<0.001 *	79.17%	<0.001 *	95.83%	<0.001 *
4 + 5 + 6 + 7	NA	0.281	NA	0.496	NA	0.677

Wilcoxon signed rank test, * *p* < 0.05. NA, the proportion of eyes with post-operative diplopia were higher than preoperative status.

**Table 5:** Univariate and multivariate regression analyses of baseline characteristics with EOM limitation and diplopia at three months postoperative.

	**Post-OP 3 M Limitation**			
	**Univariate**		**Multivariate**	
**Characteristic**	**OR (95%)**	***p* Value**	**OR (95% CI)**	***p* Value**
Duration from injury to OP (days)	1.023 (1.010–1.037)	<0.001 *	1.030 (1.015–1.048)	0.001 *
OP time (minutes)	1.010 (1.000–1.021)	0.059	1.007 (0.994–1.018)	0.267
Age (years)	0.999 (0.980–1.019)	0.928		
Gender (M/F)	2.611 (1.201–5.675)	0.015 *	2.720 (1.138–6.498)	0.024 *
Combined medial wall fracture	0.014 (0.578–1.011)	0.996		
Pre-OP limitation	2.412 (1.221–4.762)	0.011 *	2.883 (1.344–6.182)	0.007 *
	**Post-OP 3 M Diplopia**			
	**Univariate**		**Multivariate**	
**Characteristic**	**OR (95%)**	***p* Value**	**OR (95% CI)**	***p* Value**
Duration from injury to OP (days)	1.047 (1.030–1.064)	<0.001 *	1.091(1.063–1.119)	<0.001 *
OP time (minutes)	1.011 (1.002–1.020)	0.018 *	1.001(0.989–1.013)	0.269
Age (years)	0.997 (0.982–1.013)	0.745		
Gender (M/F)	2.840 (1.548–5.211)	0.001 *	3.246 (1.368–7.703)	0.008 *
Combined medial wall fracture	0.022 (0.348–1.047)	0.995		
Pre-OP diplopia	3.042 (1.774–5.217)	0.001 *	2.774 (1.496–5.145)	0.001 *

CI, Confidence Interval; EOM, extraocular movement; OP, operative; * *p* < 0.05.

## Data Availability

The data presented in this study are available on request from the corresponding author. The data are not publicly available due to privacy issue.

## Supplementary Materials

The following supporting information can be downloaded at: https://www.mdpi.com/article/10.3390/jpm12050671/s1, Table S1: Comparison of EOM limitation and diplopia among different groups before and after surgery.
