# Supplementary material for: Early Intervention in Orbital Floor Fractures: Postoperative Ocular Motility and Diplopia Outcomes
Source: J Pers Med. 2022 Apr 22;12(5):671. doi: 10.3390/jpm12050671 (PMC9144115; doi:10.3390/jpm12050671)
Supplement: Supplementary file 1 [file jpm-12-00671-s001.zip › jpm-1658795-supplementary.pdf]

Table S1. Comparison of EOM limitation and diplopia among different groups before and after surgery.

Group	EOM Limitation			Diplopia		
	P value			P value		
	Before OP vs post-OP (1wk)	Before OP vs post-OP (1m)	Before OP vs post-OP (3m)	Before OP vs post-OP (1wk)	Before OP vs post-OP (1m)	Before OP vs post-OP (3m)
1	<0.001 *	<0.001 *	<0.001 *	<0.001 *	<0.001 *	<0.001 *
2	<0.001 *	<0.001 *	<0.001 *	0.248	0.021 *	0.001 *
3	0.003 *	<0.001 *	<0.001 *	0.257	0.096	0.005 *
4	0.157	0.008 *	0.002 *	0.564	0.763	0.739
5	0.317	0.157	0.083	1.000	0.527	0.527
6	0.157	0.157	0.157	0.564	0.564	0.564
7	0.317	0.317	0.317	0.275	0.275	0.275

The Wilcoxon signed rank test was used to determine the relationship between values before and after operation; EOM, extraocular movement; OP, operation; * $P<0.05$.
